# ADAR-mediated RNA editing suppresses sleep by acting as a brake on glutamatergic synaptic plasticity

**DOI:** 10.1038/ncomms10512

**Published:** 2016-01-27

**Authors:** J. E. Robinson, J. Paluch, D. K. Dickman, W. J. Joiner

**Affiliations:** 1 Department of Pharmacology, University of California San Diego, La Jolla, California 92093-0636, USA; 2 Biomedical Sciences Graduate Program and Molecular Pharmacology Training Grant, University of California San Diego, La Jolla, California 92093-0636, USA; 3 Department of Neurobiology, University of Southern California, Los Angeles, California 90089-2520, USA; 4 Neurosciences Graduate Program, University of California San Diego, La Jolla, California 92093-0636, USA; 5 Center for Circadian Biology, University of California San Diego, La Jolla, California 92093-0636, USA

## Abstract

It has been postulated that synaptic potentiation during waking is offset by a homoeostatic reduction in net synaptic strength during sleep. However, molecular mechanisms to support such a process are lacking. Here we demonstrate that deficiencies in the RNA-editing gene *Adar* increase sleep due to synaptic dysfunction in glutamatergic neurons in *Drosophila*. Specifically, the vesicular glutamate transporter is upregulated, leading to over-activation of NMDA receptors, and the reserve pool of glutamatergic synaptic vesicles is selectively expanded in *Adar* mutants. Collectively these changes lead to sustained neurotransmitter release under conditions that would otherwise result in synaptic depression. We propose that a shift in the balance from synaptic depression towards synaptic potentiation in sleep-promoting neurons underlies the increased sleep pressure of *Adar*-deficient animals. Our findings provide a plausible molecular mechanism linking sleep and synaptic plasticity.

## Abstract (web summary)

Sleep is postulated to offset buildup in net synaptic strength that occurs during waking experience. Here, the authors identify a role for the RNA editing gene *Adar* in regulating glutamatergic synaptic plasticity and show that disruption in *Adar* expression impairs normal waking in flies.

## Graphical abstract

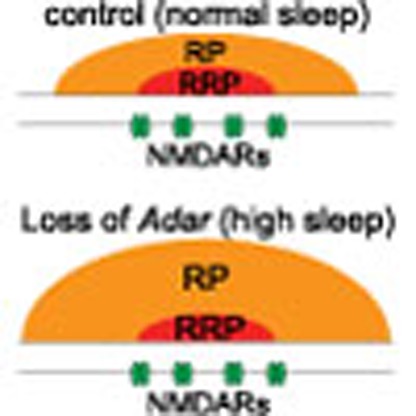

Chronically unfulfilled sleep need contributes to numerous medical problems including depression, pain, hypertension, diabetes and cardiovascular disease^1^,^2^,^3^,^4^,^5^,^6^. On a shorter timescale, even 1–2 nights of poor sleep result in attention deficits that can prove costly, or even deadly, in situations in which reaction time is critical^7^. Remarkably, despite decades of intense study, the mechanisms that control sleep need and the cellular functions that they fulfil are largely unknown. In recent years much attention has focused on the hypothesis that sleep need arises from an experience-dependent increase in net synaptic strength during waking^8^. According to the same hypothesis, sleep homeostatically reverses this increase to maintain average synaptic strength within an optimal dynamic range for synaptic plasticity. Although much experimental data support this hypothesis, criticisms persist and detailed mechanistic support for the proposed phenomenon is lacking^9^.

We set out to identify novel sleep-regulating genes in the fruit fly, *Drosophila melanogaster*, due to the genetic tractability of this organism as well as the striking parallels between sleep/wake behaviour in flies and mammals, which suggest that core functions of sleep are evolutionarily conserved. This notion has been reinforced in recent years by identification of sleep-regulating genes and signalling mechanisms in flies and mice that are believed to share similar functions, including cyclic AMP signalling, voltage-gated K^+^ channels, and dopamine among others^10^. Notably lacking, however, have been molecular discoveries relating sleep to defined forms of synaptic plasticity.

We hypothesized that if sleep is indeed functioning to maintain overall synaptic strength within a physiological range, then identifiable genes should exist that reflect the reciprocal relationship between both processes. For example, if net potentiation during waking truly drives the need to sleep, then genetic lesions resulting in increased synaptic strength should cause an increase in sleep, whereas genetic lesions resulting in decreased synaptic strength should cause a decrease in sleep. Finally, we reasoned that the resulting dysregulation should shed light on the mechanistic relation between synaptic plasticity and sleep, for which very little information is currently known.

To test these ideas we performed a genetic screen in *Drosophila* and found that the RNA-editing gene *Adar* is required for flies to maintain normal waking. Consistent with a related role in synaptic plasticity, *Adar* acts as a brake on sleep-promoting glutamatergic signalling through postsynaptic AMPA and NMDA receptors by reducing the reserve pool (RP) of synaptic vesicles available for release during sustained trains of neuronal activity. We therefore conclude that *Adar* suppresses sleep in *Drosophila* by negatively regulating short-term potentiation.

## Results

### ADAR is required for wake maintenance

**Figure 1:**
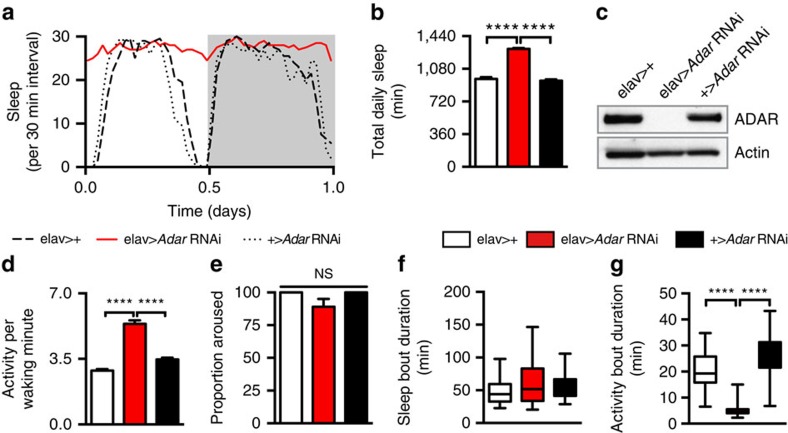
*Adar* stabilizes the waking state to suppress sleep. (**a**) Representative sleep profiles of male elav>*Adar* RNAi and controls. (**b**) Quantification of sleep in **a**. Pan-neuronal knockdown of *Adar* increases sleep in elav>*RNAi* animals relative to controls. (**c**) Representative western blot of fly brains indicates efficient knockdown of ADAR expression in elav>*Adar* RNAi flies. (**d**) Waking activity is not reduced in elav>*Adar* RNAi animals. (**e**) Sleep in elav>*Adar* RNAi animals is acutely reversible by mechanical perturbation. (**f**) Sleep maintenance is unaffected in elav>*Adar* RNAi animals. (**g**) The wake state is destabilized in elav>*Adar* RNAi animals relative to controls. For each panel: *elav*>+ (*n*=39); elav>*Adar* RNAi (*n*=54); +>*Adar* RNAi (*n*=39). For all figures *, **, *** and **** indicate *P*<0.05, 0.01, 0.001 and 0.0001, respectively, and error bars represent s.e.m.

**Figure 2:**
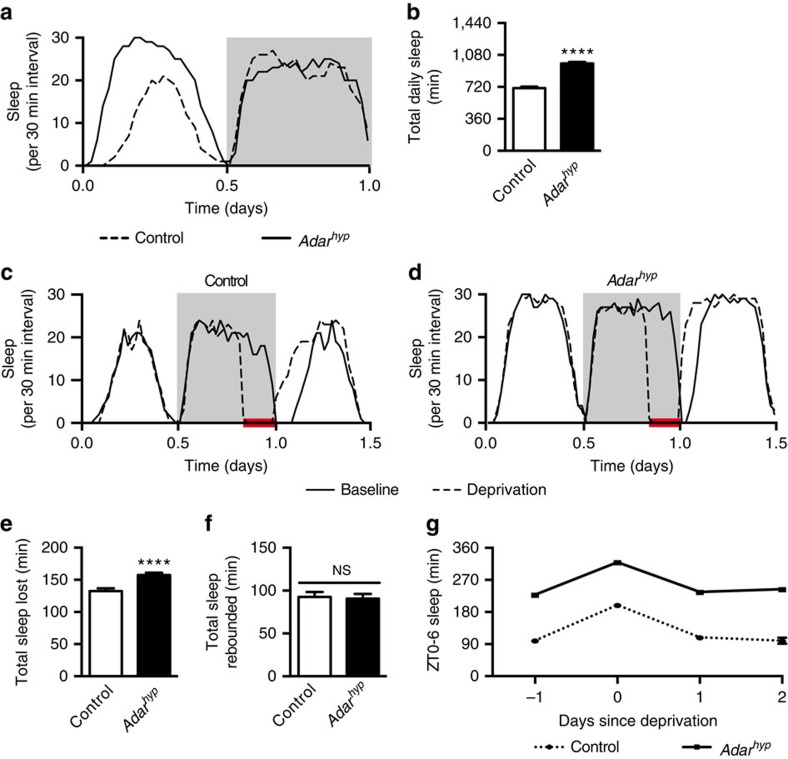
Reducing *Adar* levels does not alter sleep homeostasis. (**a** and **b**) Representative sleep profiles (**a**) and total daily sleep (**b**) for *Adar*^*hyp*^ and sibling control animals (females; *Adar*^*hyp*^ (*n*=93); control (*n*=95)). (**c** and **d**) Representative sleep profiles during baseline, deprivation (red bar; ZT20–24) and recovery periods for sibling control (**c**) and *Adar*^*hyp*^ animals (**d**). (**e** and **f**) Quantification of sleep lost during deprivation period (**e**) and recovered the next morning from ZT0–6 (**f**). (**g**) Total sleep each day from ZT0–6. Sleep returns to baseline after 1 day of recovery. (In **c**–**g**: *Adar*^*hyp*^ (*n*=90); control (*n*=95)). In this figure, control refers to siblings of *Adar*^*hyp*^ animals outcrossed into a *w*^*1118*^ genetic background. Females were used for all sleep homoeostasis experiments.

We systematically screened for neuronal genes that control sleep need in *D. melanogaster* and tested to what extent their mechanisms of action involve synaptic plasticity. Our approach involved coupling the GAL4/UAS system^11^ to RNA interference (RNAi)-dependent knockdown of genetic targets specifically in the nervous system and then assaying for effects on daily sleep. After retesting promising lines, we found that efficient knockdown of the conserved RNA-editing gene *Adar* (*Adenosine deaminase acting on RNA*) led to increased sleep in both male and female animals (*elav*>*Adar* RNAi; Fig. 1a–c; Supplementary Fig. 1a,b; Supplementary Fig. 2a), an effect that was recapitulated with a hypomorphic *Adar* allele (*Adar*^*hyp*^)^12^ that expresses just 20% of normal ADAR protein (Fig. 2a,b). Consistent with a deficit in sleep/wake control rather than in locomotion, *Adar*-deficient animals were at least as active during waking as controls (Fig. 1d; Supplementary Fig. 1c) and had inactive periods that could be fully overcome by mechanical agitation (Fig. 1e). To determine which component of sleep/wake control is regulated by *Adar*, we analysed the durations of both sleep and wake bouts and found that depletion of *Adar* selectively affected the latter, leading to destabilization of the waking state (Fig. 1f,g; Supplementary Fig. 1d,e).

Two possible explanations for the increase in sleep caused by reduction in *Adar* are altered sleep homoeostasis and increased sleep pressure. To discriminate between these possibilities we sleep-deprived *Adar* hypomorphs and control flies during the final 4 h of night and measured homoeostatic recovery sleep the next morning. We found that both groups of animals recovered ∼1.5 h of lost sleep and then returned to baseline levels in the following days (Fig. 2c–g). We interpret these data to indicate that depletion of *Adar* does not affect sleep homoeostasis. Although we acknowledge that alternative interpretations are possible if the rate of recovery of lost sleep is considered (Supplementary Fig. 3a–c), we believe the most parsimonious interpretation of our data are that *Adar* is required for normal sleep pressure, and that in *Adar* mutants this process is decoupled from compensatory homoeostatic mechanisms that would otherwise fix total daily sleep at normal levels.

### ADAR suppresses glutamatergic signalling

Previous studies in *Drosophila* have suggested that ADAR protein is expressed broadly throughout the brain^12^. To map where *Adar* functions to modulate sleep behaviour, we initially performed an anatomical screen in which we coupled UAS-*Adar* RNAi to a variety of well-characterized GAL4 drivers that express in populations of circadian clock neurons, established sleep-regulating regions of the brain, and in neurons distinguishable from one another by their distinct neurotransmitter systems (Supplementary Table 1). We also screened an additional collection of ∼500 randomly selected GAL4 lines derived from cloned putative enhancer fragments^13^. Out of both collections, the GAL4 driver 40B03 was most effective at recapitulating the increase in sleep observed with pan-neuronal knockdown of *Adar* (Supplementary Fig. 4).

As controls to confirm that knockdown of *Adar* by 40B03-*Gal4* led to increased sleep, rather than a physical disability or generalized defect in CNS function, we performed a series of additional experiments. In the first, we measured the responsiveness of knockdown animals to an arousal stimulus of fixed intensity. Consistent with the rapidly reversible nature of sleep, we found that the tendency of 40B03>*Adar* RNAi flies to remain immobile could be fully overcome by mechanical stimulation (Supplementary Fig. 5a). In a second experiment, we fed flies with the caffeine analogue, IBMX, and found that it was able to efficiently maintain waking in 40B03>*Adar* RNAi animals (Supplementary Fig. 5b). In a third experiment, we tested climbing ability and found that 40B03>*Adar* RNAi flies showed no performance deficits relative to controls (Supplementary Fig. 5c). In a fourth experiment, we tested whether increased sleep in 40B03>*Adar* RNAi animals occurs through effects on selected neuronal circuits or through a general depression of neuronal function. We reasoned that widespread effects should sensitize animals to other general CNS depressants. To test this possibility we measured the amount of time it took for flies to stop responding to a repeated mechanical stimulus in the presence of volatilized ethanol. We found that 40B03>*Adar* RNAi flies had normal sensitivity and development of tolerance to ethanol (Supplementary Fig. 5d), suggesting that *Adar* selectively modulates neural circuitry involved in sleep regulation rather than acting as a global gain control of brain activity.

**Figure 3:**
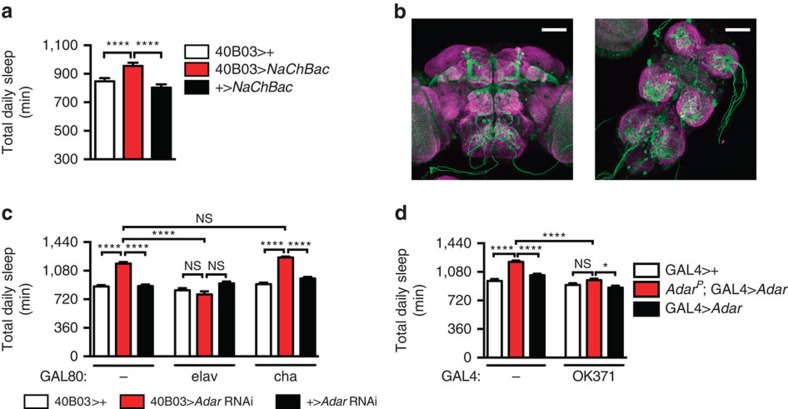
*Adar* suppresses output of glutamatergic sleep-promoting neurons. (**a**) Total daily sleep is increased in flies expressing the depolarizing NaChBac channel under control of 40B03-*Gal4* (40B03>+ (*n*=24); 40B03>*NaChBac* (*n*=30); +>*NaChBac* (*n*=31)). (**b**) Representative expression pattern of 40B03>CD8GFP (green) and of the neuropil marker, nc82 (magenta), in central brain and thoracic ganglion. Scale bars, 57 μm. (**c**) Increased sleep in 40B03>*Adar* RNAi animals is suppressed by pan-neuronal (*elav*) but not cholinergic (*cha*) expression of GAL80 (*n*=23–48 for each genotype). (**d**) Restoration of *Adar* expression in glutamatergic neurons (OK371-*Gal4*) restores sleep to control levels in *Adar* null mutants (*Adar*^*P*^; *n*=23–32 for each genotype).

We also tested the role of 40B03 neurons in regulating sleep by increasing their electrical excitability with the bacterial sodium channel, NaChBac (ref. 14). We found that 40B03>*NaChBac* flies had increased sleep compared with controls (Fig. 3a), similar to what we observed in 40B03>*Adar* RNAi animals. Our data thus suggest that 40B03 neurons promote sleep and that *Adar* suppresses output of these neurons.

To continue to address where *Adar* functions to regulate sleep, we then coupled 40B03-*Gal4* to *UAS-CD8::GFP* (40B03>*CD8GFP*) and examined whole mounts of dissected brains by confocal microscopy (Fig. 3b). Although 40B03>*CD8GFP* expression was restricted to a subset of neurons in the fly brain, we were unable to correlate specific cell types with sleep function. Notably, 40B03 expresses in a number of Kenyon cells comprising part of the mushroom bodies, which have been previously implicated in sleep^15^,^16^. However, ADAR protein does not appear to express in adult Kenyon cells^12^, and we do not observe changes in sleep after expressing *Adar* RNAi in the mushroom bodies using commonly used GAL4 drivers (Supplementary Table 1). The increased sleep we observed upon pan-neuronal knockdown of *Adar* suggested a nervous system requirement for this molecule, and indeed we also found that the neuronal suppressor of GAL4 activity, *elav-Gal80*, blocked the ability of 40B03>*Adar* RNAi to increase sleep. In contrast, the cholinergic-specific suppressor of GAL4 activity, *cha*-*Gal80*, had no effect on sleep in 40B03>*Adar* RNAi animals (Fig. 3c), suggesting a non-cholinergic role for *Adar* in sleep regulation. Thus, despite the fact that RNA editing alters the identities of thousands of transcripts^17^,^18^, our ability to map the effect of this process to non-cholinergic neurons suggested that specific cellular mechanisms might mediate *Adar*'s effects on sleep.

### Elevated DVGLUT and NMDAR activity increase sleep

Since *Adar* is known to alter synaptic transmission by unresolved mechanisms at the neuromuscular junction (NMJ)^19^, which is glutamatergic in flies, we hypothesized that *Adar*'s effects on sleep might be mediated by alterations in glutamatergic signalling in the central brain. To test this hypothesis, we compared sleep in *Adar* null mutants (*Adar*^*P*^)^20^ alone and in the presence of transgenic *Adar* expressed exclusively in glutamatergic neurons (*Adar*^*P*^;OK371>*Adar*). As expected, *Adar*^*P*^ mutants exhibited an increase in sleep, and this phenotype was significantly attenuated in *Adar*^*P*^;OK371>*Adar* animals (Fig. 3d). Thus, *Adar* expression in glutamatergic neurons is sufficient to restore nearly normal sleep to *Adar*-deficient animals.

**Figure 4:**
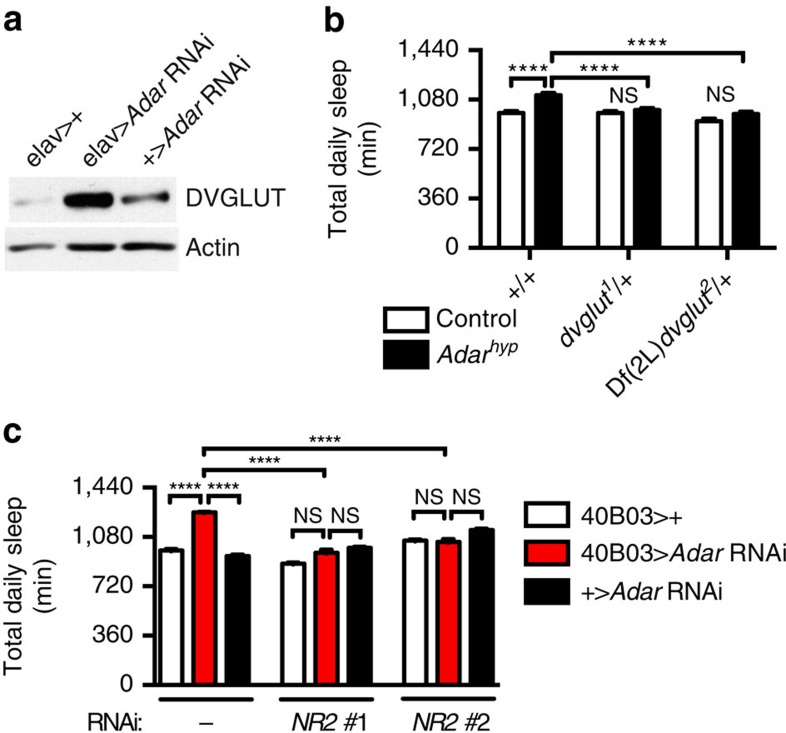
*Adar* depletion increases DVGLUT protein levels and signalling through NMDA receptors. (**a**) Representative western blot showing elevated expression of DVGLUT in heads of elav>*Adar* RNAi flies relative to controls. Actin is a loading control. (**b**) Heterozygous reduction in *dvglut* reduces sleep to control levels in *Adar*^*hyp*^ mutants (*n*=46–48 for each genotype). In this figure control refers to animals harbouring the wild-type *Adar* allele in the same *w*^*1118*^ genetic background as *Adar*^*hyp*^ mutants. (**c**) Knockdown of *NR2* restores sleep to control levels in 40B03>*Adar* RNAi animals (*n*=18–62 for each genotype).

Together with evidence that synaptic vesicles accumulate at the NMJ in *Adar* mutants^19^, these data prompted us to measure protein levels of the *Drosophila* vesicular glutamate transporter (DVGLUT) in fly heads. Consistent with a defect in synaptic signalling in central glutamatergic neurons, we found a striking increase in DVGLUT protein in flies depleted of *Adar* (Fig. 4a; Supplementary Fig. 2b). To determine whether this increase was responsible for the increase in sleep we observed in *Adar*-deficient animals, we paired *Adar* hypomorphs with a single copy of either a strong hypomorphic or a null *dvglut* allele^21^. On their own, neither of the heterozygous *dvglut* alleles affected sleep, but we found that each one was able to suppress the excess sleep of *Adar* hypomorphs (Fig. 4b). Thus, compensating for elevated DVGLUT restores normal sleep to *Adar*-deficient animals. Interestingly, even more severe reductions in *dvglut* expression led to significantly less sleep than in controls (Supplementary Fig. 6), suggesting that levels of glutamatergic signalling must be tightly maintained within a narrow range to avoid excesses and shortfalls in daily sleep.

To determine the identities of postsynaptic mediators of increased glutamatergic signalling in *Adar*-deficient animals, we knocked down various glutamate receptor transcripts in the fly genome while simultaneously reducing *Adar* expression (Supplementary Fig. 7). Interestingly, knockdown of either of two NMDA-type glutamate receptors, *NR1 or NR2*, was sufficient to partially or completely restore normal sleep to animals depleted of *Adar*. Similar results were obtained with two independent *NR2* RNAi lines (Fig. 4c). Since activation of NMDA receptors is known to require simultaneous synaptic release of glutamate and depolarizing current through AMPA-type glutamate receptors, AMPA receptor signalling would also be expected to be involved in *Adar*-dependent increases in sleep. Consistent with this expectation, we observed restoration of normal sleep to *Adar*-deficient animals on knockdown of the AMPA receptor transcript *GluRI* (Supplementary Fig. 7). Thus, *Adar* is required to reduce signalling through excitatory glutamate receptors in the brain.

### An expanded RP increases sleep in *Adar* mutants

**Figure 5:**
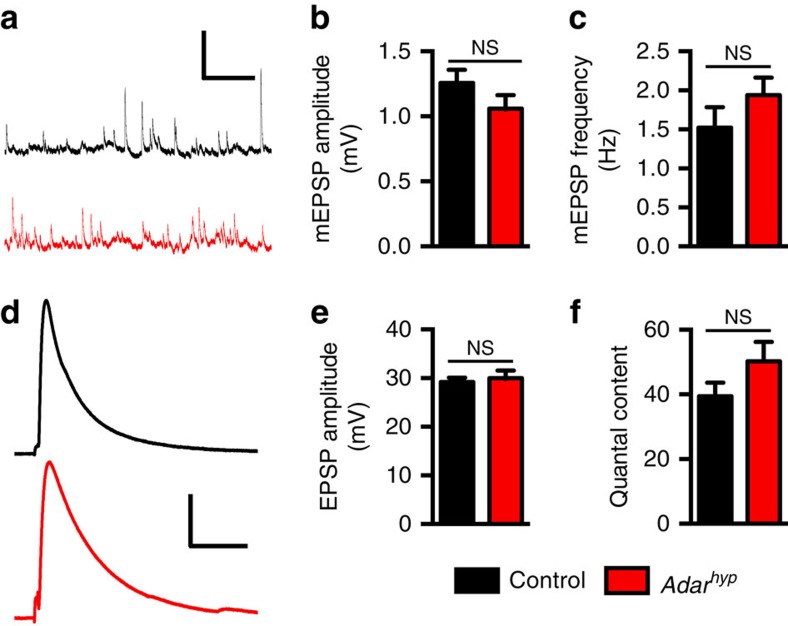
*Adar* hypomorphs have normal spontaneous and evoked synaptic transmission at the NMJ. (**a**) Representative recordings of mEPSPs in control (black) and *Adar*^*hyp*^ animals (red). Vertical and horizontal scale bars, 2 mV and 2 s, respectively. (**b** and **c**) Average mEPSP amplitudes (**b**) and frequencies (**c**) do not differ significantly between control and *Adar*^*hyp*^ animals. (**d**) Representative recordings of evoked EPSPs in control (black) and *Adar*^*hyp*^ animals (red). Vertical and horizontal scale bars, 10 mV and 50 msec, respectively. (**e** and **f**) Average evoked EPSP amplitude (**e**) and quantal content per stimulus (**f**) do not differ significantly between control and *Adar*^*hyp*^ animals (*n*=10 for control and *n*=11 for *Adar*^*hyp*^ in all panels). In this figure control refers to animals harbouring the wild-type *Adar* allele in the same *w*^*1118*^ genetic background as *Adar*^*hyp*^ mutants.

Enhanced excitatory glutamate signalling through AMPA/NMDA receptors could be achieved through several distinct mechanisms related to elevated DVGLUT expression. First, each synaptic vesicle could contain more DVGLUT, thus increasing the amount of glutamate packaged into each vesicle, which would result in an increase in quantal size^22^. In the absence of accessible and relevant neurons in the adult central nervous system from which to characterize synaptic properties, we tested this possibility by measuring spontaneous miniature excitatory postsynaptic potentials (mEPSPs) at the larval glutamatergic NMJ, which is a model for central glutamatergic synaptic transmission. In this preparation, a null allele of *Adar* has been reported to exhibit increased mEPSP amplitude^19^. However, these changes were not detectable with more moderate reductions in *Adar* that are still able to increase sleep (Figs 5a–c; 2a,b). Second, more vesicles could be released per action potential that arrives at presynaptic terminals. However, we did not measure a difference in quantal content in *Adar* hypomorphs relative to controls, as reflected in the amplitude of evoked EPSPs at the NMJs of both groups of animals (Fig. 5d–f). These data strongly suggest that reductions in *Adar* that are sufficient to increase sleep do not alter the amount of glutamate in each synaptic vesicle or the number of vesicles released per action potential during baseline synaptic transmission.

**Figure 6:**
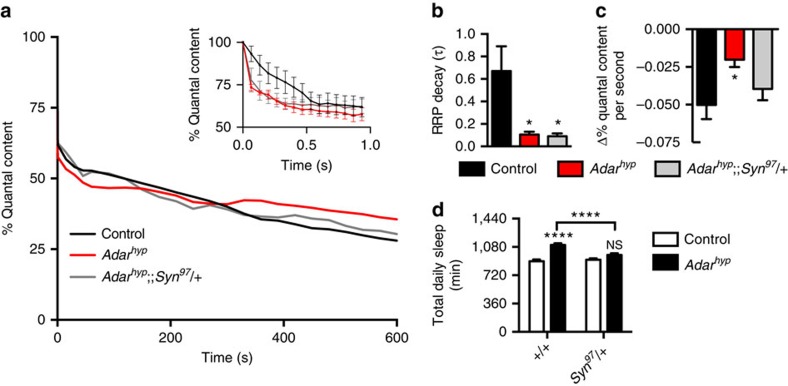
Reversing the expansion of the reserve vesicle pool restores normal sleep to *Adar* mutants. (**a**) High frequency (15 Hz) stimulation at the NMJ causes a faster rate of depletion of the RRP (inset) and a slower rate of depletion of the RP (main figure) in *Adar*^*hyp*^ mutants relative to controls. Heterozygous loss of *Synapsin* only rescues changes in the reserve pool (control (*n*=8); *Adar*^*hyp*^ (*n*=6); *Adar*^*hyp*^;;*Syn*^*97*^/+ (*n*=6)). (**b**) Quantification of the decay rate of the RRP in **a**. Tau values are based on an exponential fit to the change in quantal content over time, ending at 1.0 s (control (*n*=8); *Adar*^*hyp*^ (*n*=6); *Adar*^*hyp*^;;*Syn*^*97*^/+ (*n*=6)). (**c**) Quantification of the decay rate of the reserve pool. Values are based on linear regression fit of changes in quantal content over time, beginning at 60 s (control (*n*=8); *Adar*^*hyp*^ (*n*=6); *Adar*^*hyp*^;;*Syn*^*97*^/+ (*n*=6)). (**d**) Heterozygous loss of *Synapsin* restores normal sleep to *Adar* mutants (black bars) without altering sleep in controls (white bars; *n*=45–48 for each genotype). In **a**–**d**, control refers to animals harbouring wild-type *Adar* and wild-type *Syn* alleles in the same *w*^*1118*^ genetic background as both mutants.

These measurements also permitted us to clearly distinguish changes in synaptic vesicle size and number from potential expansions or contractions of different vesicle pools that could alternatively underlie constitutive potentiation of glutamatergic synapses. Specifically, we hypothesized that the increased sleep observed in *Adar*-depleted flies was mediated by an increase in availability of glutamatergic vesicles during sustained neuronal activity. In support of this hypothesis, a previous study reported that *Adar* mutants accumulate synaptic vesicles and vesicle-related proteins at the NMJ^19^. We also tested our hypothesis by stimulating axons at the NMJ for 10 min at 15 Hz to deplete synaptic vesicle pools while measuring the quantal content per stimulus (Fig. 6a). As described by others, we found that change in quantal content followed two temporally distinct phases: an initial period involving rapid decay followed by a subsequent period of slower, more sustained decay. Such changes have been attributed to fast and slow depletion of what are often referred to as the readily releasable pool (RRP) and RP of synaptic vesicles, respectively^23^. Intriguingly, in *Adar* hypomorphs with increased sleep we found that the fast phase decayed more quickly (Fig. 6b) and the slow phase decayed more slowly than in controls (Fig. 6c). These data are consistent with both an expanded RP in *Adar* mutants and chronically potentiated glutamatergic signalling, which could be a signal to sleep.

To determine whether our observations at the larval NMJ were relevant to altered sleep in *Adar*-deficient adults and whether a larger RP size was responsible for the increase in sleep we observed in *Adar* mutants, we focused on the possible genetic interaction between *Adar* and *Synapsin* (*Syn*). *Syn* encodes a synaptic vesicle protein that is thought to act as a barrier to transitions from the RP to the RRP^24^. A reduction in the amount of Syn should thus lower the barrier to this transition. By this logic, if increased sleep in *Adar* mutants results from an expanded glutamatergic RP, then reducing the levels of Syn should compensate for this effect. To test this hypothesis we coupled a hypomorphic mutation in *Adar* that increased sleep with a heterozygous null mutation in *Syn* (*Syn*^*97*^; ref. 25) and measured rates of depletion of the RP and RRP during high-frequency presynaptic stimulation of the NMJ. Consistent with our hypothesis, the rate of depletion of the RP was restored nearly to control levels in *Adar*^*hyp*^;;*Syn*^*97*^/+ double mutants without altering the rate of depletion of the RRP (Fig. 6b,c). We then reasoned that if an expanded glutamatergic RP indeed increases sleep need, then restoring the size of the RP to normal by reducing levels of *Syn* should also restore normal levels of sleep to *Adar* mutants. To test this hypothesis, we measured total daily sleep in animals containing *Adar*^*hyp*^ alone or in combination with the heterozygous *Syn*^*97*^ allele. Consistent with our hypothesis, sleep was fully restored to control levels in *Adar*^*hyp*^;;*Syn*^*97*^/+ mutants. Importantly, sleep was not reduced by heterozygous *Syn*^*97*^ alone, thus demonstrating a specific genetic interaction between *Adar* and *Syn* (Fig. 6d). Together, these data strongly suggest that *Adar* normally restricts the size of the RP, thus reducing glutamatergic synaptic potentiation via downstream NMDAR signalling, which in turn limits sleep pressure. In the absence of *Adar*, this brake on synaptic potentiation appears to be released in sleep-promoting neurons, thus leading to increased sleep pressure.

## Discussion

By describing a role for *Adar* in suppressing sleep, we have discovered a mechanistic link between sleep and synaptic plasticity. Such a link has been hypothesized for many years but has lacked molecular details to support it^8^,^9^. To arrive at this link, we initially identified *Adar* as a sleep-regulating gene through a genetic screen, mapped the hypersomnolence of *Adar* mutants to sleep-promoting glutamatergic neurons, showed that this phenotype was associated with an increase in DVGLUT, and found that it could be compensated by genetic reductions in *dvglut*, *NR1*, *NR2* and *GluRI*. Collectively, these data suggest that *Adar* normally suppresses glutamatergic signalling to reduce sleep.

On the basis of our results, this signalling pathway appears to be at least as important as other arousal systems in regulating sleep. For example, previous reports have shown that overall dopamine, octopamine and acetylcholine promote waking in flies^26^,^27^,^28^,^29^,^30^,^31^, whereas serotonin predominantly promotes sleep^32^. Similar to these neurotransmitters, we found that availability of glutamate correlates with arousal state, wherein elevated glutamate promotes sleep and reduced glutamate leads to increased waking. Thus, it is likely that glutamatergic signalling is tightly regulated to maintain proper levels and timing of sleep—a process in which we have now implicated *Adar*.

In elucidating the pathway through which *Adar* acts to regulate sleep, we found two points at which synaptic plasticity might be implicated. The first is based on our observation that the sleep-promoting effects of *Adar* mutants require AMPA and NMDA receptors. NMDA receptors are frequently involved in synaptic plasticity^33^,^34^,^35^, making them prime candidates for potentiated synaptic responses that have been proposed to accumulate during waking and in turn drive compensatory sleep need. In fact, a recent report has described *NR1* as a novel sleep-promoting gene in *Drosophila*^36^. Here we have confirmed that NMDA receptors promote sleep, and we have demonstrated that they are required for the sleep phenotype of *Adar* mutants. Thus, it is likely that a major role of wild-type *Adar* is to act as a presynaptic brake on NMDA-dependent postsynaptic potentiation.

We were able to more thoroughly investigate the second mechanism by which *Adar* appears to regulate glutamatergic synaptic plasticity to influence sleep. In this case, we found that basal synaptic transmission in glutamatergic neurons was not altered by depletion of *Adar*. That is, *Adar* hypomorphs had normal glutamate loading into synaptic vesicles, postsynaptic responses to spontaneously released glutamate, and number of glutamatergic vesicles released per evoked excitatory postsynaptic potential. However, upon sustained stimulation, *Adar* mutants exhibited decreased synaptic depression that could be compensated by reducing *Syn*, a gene that limits depletion of the RP^24^. Notably, reducing *Syn* was also sufficient to reduce sleep in *Adar* mutants to control levels. Our data thus support the hypothesis that *Adar* mutants have an expanded synaptic RP, which reduces depression of sleep-promoting glutamatergic neurons to increase intrusions of sleep into the waking state.

Expansion of the RP of synaptic vesicles, like those we have observed in *Adar* mutants, has been shown to substantially increase the amount of information transmitted per burst of action potentials due to decreased short-term depression^37^. In essence, this phenomenon shifts the balance of depression and potentiation towards the latter. We suggest that in glutamatergic sleep-promoting neurons, this shift translates to an increased probability of sleep onset from spike trains that would normally be sub-threshold for the behaviour.

Although our study fleshes out mechanistic details linking modulation of short-term plasticity to sleep, hints of such a connection exist from other studies as well. For example, loss-of-function mutations in the Fragile X mental retardation 1 (*Fmr1*) gene cause an expansion of total synaptic vesicles in mice^38^ and an increase in sleep in *Drosophila*^39^. Conversely, loss of calcineurin impairs synaptic vesicle recycling in mice, resulting in enhanced synaptic depression^40^, and causes reduced sleep in *Drosophila*^41^,^42^. Although these effects and our own findings are consistent with a positive correlation between short-term synaptic potentiation and sleep, other findings suggest a more complex relationship. For example, loss of *Rab3a* in mice reduces the probability of synaptic vesicle release following a train of action potentials^43^, thereby increasing synaptic depression, and increases non-REM sleep^44^. In addition, loss of the Rab3-interacting molecule *RIM1α* reduces presynaptic calcium influx in mice, thereby increasing short-term facilitation^45^, and reduces REM sleep^46^. *Rab3a* and *RIM1α* mutant mice have other phenotypes as well, thus complicating the establishment of a clear relationship between short-term plasticity and sleep. Nonetheless, although the directionality of change for each phenomenon may depend on which neural circuits are impacted, there is ample evidence linking short-term plasticity and sleep in various genetic perturbations. Our own findings underscore this idea.

It will be interesting to determine if similar mechanisms link sleep to potentiating responses involved in other forms of behavioural plasticity. The link between RNA editing and glutamatergic plasticity that we have described may be particularly fruitful to explore in other contexts as well. Hints of such a relationship have been described previously. For example, early observations of the role of RNA editing in the brain showed that ADAR functions postsynaptically to limit Ca^2+^ permeability and channel conductance of GluR2-containing receptors^47^,^48^. Furthermore, editing of GluR2, GluR3 and GluR4 at the R/G position has been shown to alter receptor gating kinetics, resulting in more rapid desensitization and recovery from desensitization^49^. Together with evidence that RNA editing is reduced in glutamate excitotoxic diseases such as ALS^50^,^51^,^52^, we suggest that a major function of RNA editing in the nervous system is to limit glutamatergic signalling. Since we have shown that ADAR acts as a presynaptic glutamatergic brake in flies, it will be interesting to determine whether this function is conserved in mammalian nervous systems and whether ADAR is also able to achieve the same effect through postsynaptic regulatory mechanisms.

## Methods

### Fly stocks

*D. melanogaster* were grown at room temperature (20–22 °C) on standard cornmeal media with yeast. Unless otherwise indicated, all animals were outcrossed a minimum of 5 times into a *w*^*1118*^ *iso31* genetic background. *Adar*^*HA*^ and *Adar*^*hyp*^ were obtained from Dr. Robert Reenan. *Adar*^*P*^ and *UAS-Adar* were obtained from Dr. Gabriel Haddad. *dvglut*^*1*^ and *Df(2L)dvglut*^*2*^ were obtained from Dr. Aaron DiAntonio. The *Adar* RNAi line, *7764*, was obtained from the Vienna Drosophila Resource Center ( http://stockcenter.vdrc.at/control/main) and was used in the presence of *UAS-Dicer* in all experiments. *UAS-CD8::GFP*, *UAS-Dicer* (second and third chromosome insertions), *elav-Gal4*, *dvglut*^*MI02805*^, *OK371-Gal4*, *Syn*^*97*^, *NR1* RNAi (HMS02200), *NR2* #1 RNAi (HMS02012), *NR2* #2 RNAi (HMS02176), *GluRI* RNAi (HMS02155), *GluRIB* RNAi (JF02752), *GluRIIA* RNAi (JF03145), *GluRIIB* RNAi (JF03145), *GluRIIC* RNAi (JF01854), *GluRIID* RNAi (JF02035), *GluRIIE* RNAi (JF01962), *Clumsy* RNAi (JF02987), *CG3822* RNAi (JF01873), *CG5621* RNAi (JF01840) and *CG11155* RNAi (JF03425), *mGluRA* RNAi (JF01958; refs 53, 54), 40B03-*Gal4* and all other *Gal4* lines^13^ were obtained from the Bloomington Stock Center ( http://flystocks.bio.indiana.edu/). Male flies were used for all behavioural assays unless otherwise indicated.

### Sleep and locomotor measurements

Individual 3–7-days old flies were placed in 5 × 65-mm Pyrex tubes containing a mixture of 2% agarose and 5% sucrose at one end as a food source. Animals were entrained for 2 days in 12 h:12 h light:dark conditions at 25 °C, and infrared beam breaks were recorded in 1 min bins for the next 2–4 days using the *Drosophila* Activity Monitoring System (Trikinetics). Sleep analysis was performed as previously described using custom MATLAB (Mathworks) software^30^. For IBMX treatment, 1 day of baseline sleep was recorded on standard food before animals were moved at ZT0 into new glass tubes containing food supplemented with 0.1 mg ml^−1^ IBMX (Sigma). Sleep was calculated for the second day on drug.

### Sleep deprivation and rebound

Flies in DAM2 *Drosophila* activity monitors (Trikinetics) were sleep deprived by periodic shaking in a VX-2500 multi-tube vortexer (VWR) for 2 s min^−1^ from ZT20–24. Animals were deprived of sleep for 4 h because this duration was found to be sufficient to elicit saturating levels of rebound without physically damaging animals. Rebound sleep was measured as immediate post-deprivation minus pre-deprivation sleep at ZT0–6.

### Sleep reversibility measurements

Flies were loaded into DAM2 activity monitors and placed on a custom built platform that oscillated along a line perpendicular to the axis of locomotion. Oscillation speed and timing were controlled using an Arduino Uno-powered motor. Arousal stimuli consisted of 10 s oscillations every minute for 5 min at ZT0–6. Sleep was considered to be reversible for any fly that moved within 5 min of the final stimulation. Dead flies were also included as negative controls to rule out spurious detection of activity due to involuntary motion across the infrared beam.

### Climbing assays

Three to seven-day old flies were transferred at ZT2 to two empty vials (25 × 95 mm) connected to each other vertically at their open ends. After 10 min acclimation, flies were tapped to the bottom of the lower vial. After 30 s a divider was placed between the vials and the percentage of flies that successfully climbed into the upper vial was measured. Means for each genotype were recorded after three trials.

### Ethanol sensitivity and tolerance

Three to seven- day old flies were transferred at ZT2 to empty vials and tested every minute for loss-of-righting reflex during exposure to ethanol as previously described^55^. The time for 50% of the animals to remain stationary (ST50) was measured. Continued exposure to ethanol was maintained for approximately twice this time before transferring animals to a fresh vial. After 4 h recovery, the same animals were transferred back to ethanol-containing vials, and ST50 was measured again to determine ethanol tolerance.

### Immunohistochemistry

Immunohistochemistry was performed as described previously^30^. Briefly, 3–7-day old brains were dissected in ice-cold PBS, fixed in 4% paraformaldehyde, and blocked in PBST (PBS and 0.3% Triton X-100) with 5% normal donkey serum (Jackson Laboratory) before staining. Brains were then incubated with 1:1,000 rabbit anti-GFP (Invitrogen) and 1:50 mouse anti-nc82 (Developmental Studies Hybridoma Bank) antibodies overnight at 4 °C and washed five times in PBST. Brains were then incubated with 1:1,000 Alexa 568 anti-rabbit (Life Technologies) and 1:1,000 Alexa 633 anti-mouse (Life Technologies) antibodies for 4 h at room temperature before washing five times in PBST and coverslip mounting in Vectashield (Vector Labs). Images were taken at × 40 magnification on a Leica SP5 confocal microscope at 0.5 mm intervals and reassembled for display as maximum projections using Fiji^56^.

### Western blot analysis

Brains or heads (15–20) were dissected from 3–7-day old flies and lysed in sample buffer (20 mM HEPES, pH 7.5, 100 mM KCl, 10 mM EDTA, 50 mM NaF, 0.1% Triton X-100, 10% glycerol, 1 mM DTT, 1X Roche Complete Protease Inhibitors). Lysates were cleared of particulate debris by centrifugation at 5,000*g* for 5 min at 4 °C before protein quantification. Lysates were resolved on 10-wells 4–12% NuPAGE SDS-PAGE gels (Invitrogen) and transferred to nitrocellulose membranes. Membranes were probed using 1:500 mouse anti-HA (Covance), 1:10,000 rabbit anti-DVGLUT^22^ and 1:10,000 mouse anti-actin primary antibodies (EMD Millipore) followed by 1:5,000 anti-mouse, 1:5,000 anti-rabbit and 1:10,000 anti-mouse HRP antibodies, respectively (VWR). Visualization of bound secondary antibodies was achieved using SuperSignal West Pico Chemiluminescent Substrate (Thermo Scientific). Images in the main figures have been cropped for presentation. Full size images are presented in Supplementary Fig. 2.

### Electrophysiology

All dissections and recordings were performed in modified HL3 saline^57^ containing (in mM): 70 NaCl, 5 KCl, 10 MgCl2, 10 NaHCO_3_, 115 Sucrose, 5 Trehelose, 5 HEPES and 0.4 CaCl_2_ (unless otherwise specified), pH 7.2. Neuromuscular junction sharp electrode (electrode resistance between 10 and 35 MΩ) recordings were performed on muscles 6 and 7 of abdominal segments A2 and A3 in wandering third instar larvae. Larvae were cultured in standard molasses medium, raised at 25 °C and dissected; the guts, trachea and ventral nerve cord were removed from the larval body walls with the motor nerve carefully cut, and the preparation was rinsed several times with HL3 saline. Recordings were performed on an Olympus BX51 WI microscope using a × 40/0.80 water-dipping objective. Recordings were acquired using Axoclamp 900A amplifier, Digidata 1440A acquisition system and pClamp 10.5 software (Molecular Devices). Electrophysiological sweeps were digitized at 10 kHz, and filtered at 1 kHz. Data were analysed using Clampfit (Molecular devices), MiniAnalysis (Synaptosoft) and Excel (Microsoft).

mEPSPs were recorded in the absence of any stimulation, and cut motor axons were stimulated at ∼5 nA for 3 ms to elicit EPSPs. To fine tune stimulus intensity, an ISO-Flex stimulus isolator was used (A.M.P.I.). Intensity was adjusted for each cell, set high enough to consistently elicit full responses in both axons innervating the muscle segment. Average mEPSP amplitude, EPSP amplitude, and quantal content were calculated for each genotype with corrections for nonlinear summation^58^. Muscle input resistance (R_in_) and resting membrane potential (V_rest_) were monitored during each experiment. Recordings were rejected if the V_rest_ was more depolarized than −60 mV, if the R_in_ was less than 5MΩ, or if either measurement deviated by more than 10% during the experiment. For synaptic vesicle rundown experiments, postsynaptic responses for each preparation were fit to a single exponential curve RRP or averaged into 30 s bins and fit from 60 s to 9 min by linear regression (RP).

### Data analysis and statistics

Replicates (*n* values) represent the number of biological replicates for each experimental condition. Bar graphs depict the mean±s.e.m., except for box and whisker plots, which depict the median (line), 25th to 75th percentiles (box) and minimum/maximum values (whiskers). Unless otherwise indicated, datasets that approximate a normal Gaussian distribution were analysed with unpaired Student's *t*-test followed by Welch's correction for comparisons between two groups. For experiments of single factor design, we analysed data using one-way analysis of variance followed by the Tukey–Kramer test for multiple comparisons or Sidak's multiple comparisons test for select comparisons. Two-way analysis of variance followed by Sidak's multiple comparisons test was used to analyse experiments of two-factor design. For datasets from non-Gaussian distributions, comparisons were performed using Kruskal–Wallis test followed by Dunn's multiple comparisons test. All statistical tests were two-sided and performed using Prism 6.0 f for Mac OS X (GraphPad Software).

## Additional information

**How to cite this article:** Robinson, J. E. *et al*. ADAR-mediated RNA editing suppresses sleep by acting as a brake on glutamatergic synaptic plasticity. *Nat. Commun.* 7:10512 doi: 10.1038/ncomms10512 (2016).

## Supplementary Material

Supplementary Information Supplementary Figures 1-7 and Supplementary Table 1

## References

[b1] Mullington J. M., Haack M., Toth M., Serrador J. M. & Meier-Ewert H. K. Cardiovascular, inflammatory, and metabolic consequences of sleep deprivation. Prog. Cardiovasc. Dis. 51, 294–302 (2009). 19110131 10.1016/j.pcad.2008.10.003 PMC3403737

[b2] Johnson E. O., Roth T. & Breslau N. The association of insomnia with anxiety disorders and depression: exploration of the direction of risk. J. Psychiatr. Res. 40, 700–708 (2006). 16978649 10.1016/j.jpsychires.2006.07.008

[b3] Gangwisch J. E. . Sleep duration as a risk factor for diabetes incidence in a large U.S. sample. Sleep 30, 1667–1673 (2007). 18246976 10.1093/sleep/30.12.1667 PMC2276127

[b4] Gangwisch J. E. . Short sleep duration as a risk factor for hypertension: analyses of the first National Health and Nutrition Examination Survey. Hypertension 47, 833–839 (2006). 16585410 10.1161/01.HYP.0000217362.34748.e0

[b5] Gottlieb D. J. . Association of sleep time with diabetes mellitus and impaired glucose tolerance. Arch. Intern. Med. 165, 863–867 (2005). 15851636 10.1001/archinte.165.8.863

[b6] Ayas N. T. . A prospective study of sleep duration and coronary heart disease in women. Arch. Intern. Med. 163, 205–209 (2003). 12546611 10.1001/archinte.163.2.205

[b7] McCoy J. G. & Strecker R. E. The cognitive cost of sleep lost. Neurobiol. Learn. Mem. 96, 564–582 (2011). 21875679 10.1016/j.nlm.2011.07.004 PMC3614362

[b8] Tononi G. & Cirelli C. Sleep and the price of plasticity: from synaptic and cellular homeostasis to memory consolidation and integration. Neuron 81, 12–34 (2014). 24411729 10.1016/j.neuron.2013.12.025 PMC3921176

[b9] Frank M. G. Erasing synapses in sleep: is it time to be SHY? Neural. Plast. 2012, 264378 (2012). 22530156 10.1155/2012/264378 PMC3317003

[b10] Sehgal A. & Mignot E. Genetics of sleep and sleep disorders. Cell 146, 194–207 (2011). 21784243 10.1016/j.cell.2011.07.004 PMC3153991

[b11] Brand A. H. & Perrimon N. Targeted gene expression as a means of altering cell fates and generating dominant phenotypes. Development 118, 401–415 (1993). 8223268 10.1242/dev.118.2.401

[b12] Jepson J. E. . Engineered alterations in RNA editing modulate complex behavior in *Drosophila*: regulatory diversity of adenosine deaminase acting on RNA (ADAR) targets. J. Biol. Chem. 286, 8325–8337 (2011). 21078670 10.1074/jbc.M110.186817 PMC3048717

[b13] Jenett A. . A GAL4-driver line resource for *Drosophila* neurobiology. Cell Rep. 2, 991–1001 (2012). 23063364 10.1016/j.celrep.2012.09.011 PMC3515021

[b14] Nitabach M. N. Electrical hyperexcitation of lateral ventral pacemaker neurons desynchronizes downstream circadian oscillators in the fly circadian circuit and induces multiple behavioral periods. J. Neurosci. 26, 479–489 (2006). 16407545 10.1523/JNEUROSCI.3915-05.2006 PMC2597197

[b15] Joiner W. J., Crocker A., White B. & Sehgal A. Sleep in *Drosophila* is regulated by adult mushroom bodies. Nature 441, 757–760 (2006). 16760980 10.1038/nature04811

[b16] Pitman J. L., McGill J. J., Keegan K. P. & Allada R. A dynamic role for the mushroom bodies in promoting sleep in *Drosophila*. Nature 441, 753–756 (2006). 16760979 10.1038/nature04739

[b17] St Laurent G. . Genome-wide analysis of A-to-I RNA editing by single-molecule sequencing in *Drosophila*. Nat. Struct. Mol. Biol. 20, 1333–1339 (2013). 24077224 10.1038/nsmb.2675

[b18] Graveley B. R. . The developmental transcriptome of *Drosophila melanogaster*. Nature 471, 473–479 (2011). 21179090 10.1038/nature09715 PMC3075879

[b19] Maldonado C., Alicea D., Gonzalez M., Bykhovskaia M. & Marie B. Adar is essential for optimal presynaptic function. Mol. Cellular Neurosci. 52, 173–180 (2012). 10.1016/j.mcn.2012.10.009 PMC3613243 23127996

[b20] Ma E., Gu X. Q., Wu X., Xu T. & Haddad G. G. Mutation in pre-mRNA adenosine deaminase markedly attenuates neuronal tolerance to O2 deprivation in *Drosophila melanogaster*. J. Clin. Invest. 107, 685–693 (2001). 11254668 10.1172/JCI11625 PMC208948

[b21] Daniels R. W. . A single vesicular glutamate transporter is sufficient to fill a synaptic vesicle. Neuron 49, 11–16 (2006). 16387635 10.1016/j.neuron.2005.11.032 PMC2248602

[b22] Daniels R. W. . Increased expression of the *Drosophila* vesicular glutamate transporter leads to excess glutamate release and a compensatory decrease in quantal content. J. Neurosci. 24, 10466–10474 (2004). 15548661 10.1523/JNEUROSCI.3001-04.2004 PMC6730318

[b23] Kuromi H. & Kidokoro Y. Two distinct pools of synaptic vesicles in single presynaptic boutons in a temperature-sensitive *Drosophila* mutant, shibire. Neuron 20, 917–925 (1998). 9620696 10.1016/s0896-6273(00)80473-0

[b24] Akbergenova Y. & Bykhovskaia M. Synapsin maintains the reserve vesicle pool and spatial segregation of the recycling pool in *Drosophila* presynaptic boutons. Brain Res. 1178, 52–64 (2007). 17904536 10.1016/j.brainres.2007.08.042

[b25] Godenschwege T. A. . Flies lacking all synapsins are unexpectedly healthy but are impaired in complex behaviour. Eur. J. Neurosci. 20, 611–622 (2004). 15255973 10.1111/j.1460-9568.2004.03527.x

[b26] Kume K., Kume S., Park S. K., Hirsh J. & Jackson F. R. Dopamine is a regulator of arousal in the fruit fly. J. Neurosci. 25, 7377–7384 (2005). 16093388 10.1523/JNEUROSCI.2048-05.2005 PMC6725300

[b27] Andretic R., van Swinderen B. & Greenspan R. J. Dopaminergic modulation of arousal in *Drosophila*. Curr. Biol. 15, 1165–1175 (2005). 16005288 10.1016/j.cub.2005.05.025

[b28] Crocker A. & Sehgal A. Octopamine regulates sleep in *Drosophila* through protein kinase A-dependent mechanisms. J. Neurosci. 28, 9377–9385 (2008). 18799671 10.1523/JNEUROSCI.3072-08a.2008 PMC2742176

[b29] Crocker A., Shahidullah M., Levitan I. B. & Sehgal A. Identification of a neural circuit that underlies the effects of octopamine on sleep:wake behavior. Neuron 65, 670–681 (2010). 20223202 10.1016/j.neuron.2010.01.032 PMC2862355

[b30] Wu M., Robinson J. E. & Joiner W. J. SLEEPLESS is a bifunctional regulator of excitability and cholinergic synaptic transmission. Curr. Biol. 24, 621–629 (2014). 24613312 10.1016/j.cub.2014.02.026 PMC4059605

[b31] Shi M., Yue Z., Kuryatov A., Lindstrom J. M. & Sehgal A. Identification of Redeye, a new sleep-regulating protein whose expression is modulated by sleep amount. Elife 3, e01473 (2014). 24497543 10.7554/eLife.01473 PMC3912633

[b32] Yuan Q., Joiner W. J. & Sehgal A. A sleep-promoting role for the *Drosophila* serotonin receptor 1A. Curr. Biol. 16, 1051–1062 (2006). 16753559 10.1016/j.cub.2006.04.032

[b33] Tsien J. Z., Huerta P. T. & Tonegawa S. The essential role of hippocampal CA1 NMDA receptor-dependent synaptic plasticity in spatial memory. Cell 87, 1327–1338 (1996). 8980238 10.1016/s0092-8674(00)81827-9

[b34] Liu L. . Role of NMDA receptor subtypes in governing the direction of hippocampal synaptic plasticity. Science 304, 1021–1024 (2004). 15143284 10.1126/science.1096615

[b35] Zhao M. G. . Roles of NMDA NR2B subtype receptor in prefrontal long-term potentiation and contextual fear memory. Neuron 47, 859–872 (2005). 16157280 10.1016/j.neuron.2005.08.014

[b36] Tomita J., Ueno T., Mitsuyoshi M., Kume S. & Kume K. The NMDA receptor promotes sleep in the fruit fly, *Drosophila melanogaster*. PLoS ONE 10, e0128101 (2015). 26023770 10.1371/journal.pone.0128101 PMC4449117

[b37] Rotman Z., Deng P.-Y. & Klyachko V. A. Short-term plasticity optimizes synaptic information transmission. J. Neurosci. 31, 14800–14809 (2011). 21994397 10.1523/JNEUROSCI.3231-11.2011 PMC6703406

[b38] Deng P.-Y., Sojka D. & Klyachko V. A. Abnormal Presynaptic Short-Term Plasticity and Information Processing in a Mouse Model of Fragile X Syndrome. J. Neurosci. 31, 10971–10982 (2011). 21795546 10.1523/JNEUROSCI.2021-11.2011 PMC6623101

[b39] Bushey D., Tononi G. & Cirelli C. The *Drosophila* fragile X mental retardation gene regulates sleep need. J. Neurosci. 29, 1948–1961 (2009). 19228950 10.1523/JNEUROSCI.4830-08.2009 PMC2750079

[b40] Cottrell J. R. . Working memory impairment in calcineurin knock-out mice is associated with alterations in synaptic vesicle cycling and disruption of high-frequency synaptic and network activity in prefrontal cortex. J. Neurosci. 33, 10938–10949 (2013). 23825400 10.1523/JNEUROSCI.5362-12.2013 PMC3718364

[b41] Nakai Y. . Calcineurin and its regulator Sra/DSCR1 are essential for sleep in *Drosophila*. J. Neurosci. 31, 12759–12766 (2011). 21900555 10.1523/JNEUROSCI.1337-11.2011 PMC6623415

[b42] Tomita J. . Pan-neuronal knockdown of calcineurin reduces sleep in the fruit fly, *Drosophila melanogaster*. J. Neurosci. 31, 13137–13146 (2011). 21917797 10.1523/JNEUROSCI.5860-10.2011 PMC6623252

[b43] Schluter O. M., Basu J., Sudhof T. C. & Rosenmund C. Rab3 superprimes synaptic vesicles for release: implications for short-term synaptic plasticity. J. Neurosci. 26, 1239–1246 (2006). 16436611 10.1523/JNEUROSCI.3553-05.2006 PMC6674574

[b44] Kapfhamer D. . Mutations in Rab3a alter circadian period and homeostatic response to sleep loss in the mouse. Nat. Genet. 32, 290–295 (2002). 12244319 10.1038/ng991

[b45] Kintscher M., Wozny C., Johenning F. W., Schmitz D. & Breustedt J. Role of RIM1alpha in short- and long-term synaptic plasticity at cerebellar parallel fibres. Nat. Commun. 4, 2392 (2013). 23999086 10.1038/ncomms3392

[b46] Lonart G., Tang X., Simsek-Duran F., Machida M. & Sanford L. D. The role of active zone protein Rab3 interacting molecule 1 alpha in the regulation of norepinephrine release, response to novelty, and sleep. Neuroscience 154, 821–831 (2008). 18495360 10.1016/j.neuroscience.2008.03.047

[b47] Burnashev N., Monyer H., Seeburg P. H. & Sakmann B. Divalent ion permeability of AMPA receptor channels is dominated by the edited form of a single subunit. Neuron 8, 189–198 (1992). 1370372 10.1016/0896-6273(92)90120-3

[b48] Swanson G. T., Kamboj S. K. & Cull-Candy S. G. Single-channel properties of recombinant AMPA receptors depend on RNA editing, splice variation, and subunit composition. J. Neurosci. 17, 58–69 (1997). 8987736 10.1523/JNEUROSCI.17-01-00058.1997 PMC6793687

[b49] Lomeli H. . Control of kinetic properties of AMPA receptor channels by nuclear RNA editing. Science 266, 1709–1713 (1994). 7992055 10.1126/science.7992055

[b50] Kawahara Y. . Glutamate receptors: RNA editing and death of motor neurons. Nature 427, 801 (2004). 14985749 10.1038/427801a

[b51] Hideyama T. . Induced loss of ADAR2 engenders slow death of motor neurons from Q/R site-unedited GluR2. J. Neurosci. 30, 11917–11925 (2010). 20826656 10.1523/JNEUROSCI.2021-10.2010 PMC6633551

[b52] Yamashita T. . Rescue of amyotrophic lateral sclerosis phenotype in a mouse model by intravenous AAV9-ADAR2 delivery to motor neurons. EMBO Mol. Med. 5, 1710–1719 (2013). 24115583 10.1002/emmm.201302935 PMC3840487

[b53] Ni J.-Q. . A *Drosophila* resource of transgenic RNAi Lines for Neurogenetics. Genetics 182, 1089–1100 (2009). 19487563 10.1534/genetics.109.103630 PMC2728850

[b54] Ni J. Q. . A genome-scale shRNA resource for transgenic RNAi in *Drosophila*. Nat. Methods 8, 405–407 (2011). 21460824 10.1038/nmeth.1592 PMC3489273

[b55] Maples T. & Rothenfluh A. A simple way to measure ethanol sensitivity in flies. J. Vis. Exp. 48, e2541 (2011). 10.3791/2541 PMC3339835 21372791

[b56] Schindelin J. . Fiji: an open-source platform for biological-image analysis. Nat. Methods 9, 676–682 (2012). 22743772 10.1038/nmeth.2019 PMC3855844

[b57] Dickman D. K. & Davis G. W. The schizophrenia susceptibility gene dysbindin controls synaptic homeostasis. Science 326, 1127–1130 (2009). 19965435 10.1126/science.1179685 PMC3063306

[b58] Martin A. R. A further study of the statistical composition on the end-plate potential. J. Physiol. 130, 114–122 (1955). 13278890 10.1113/jphysiol.1955.sp005397 PMC1363457

