# Supplementary material for: ADAR-mediated RNA editing suppresses sleep by acting as a brake on glutamatergic synaptic plasticity
Source: Nat Commun. 2016 Jan 27;7:10512. doi: 10.1038/ncomms10512 (PMC4737855; doi:10.1038/ncomms10512)
Supplement: Supplementary Information — Supplementary Figures 1-7 and Supplementary Table 1 [file ncomms10512-s1.pdf]

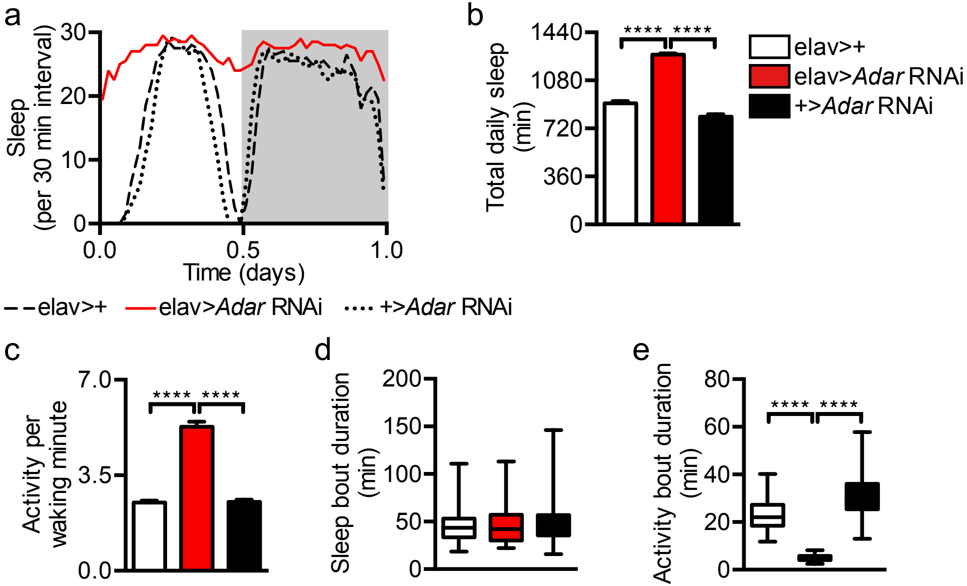

Supplementary Figure 1. Reducing *Adar* expression in female flies increases sleep.
(a) Representative sleep profiles of female elav>*Adar* RNAi and controls. (b) Quantification of sleep in (a). Pan-neuronal knockdown of *Adar* increases sleep in elav>*Adar* RNAi animals relative to controls. (c) Waking activity is not reduced in elav>*Adar* RNAi females. (d) Sleep maintenance is unaffected in elav>*Adar* RNAi females. (e) The wake state is destabilized in elav>*Adar* RNAi females relative to controls. For all panels, elav>*Adar* RNAi (n=37); elav>+ (n=63); +>*Adar* RNAi (n=40).

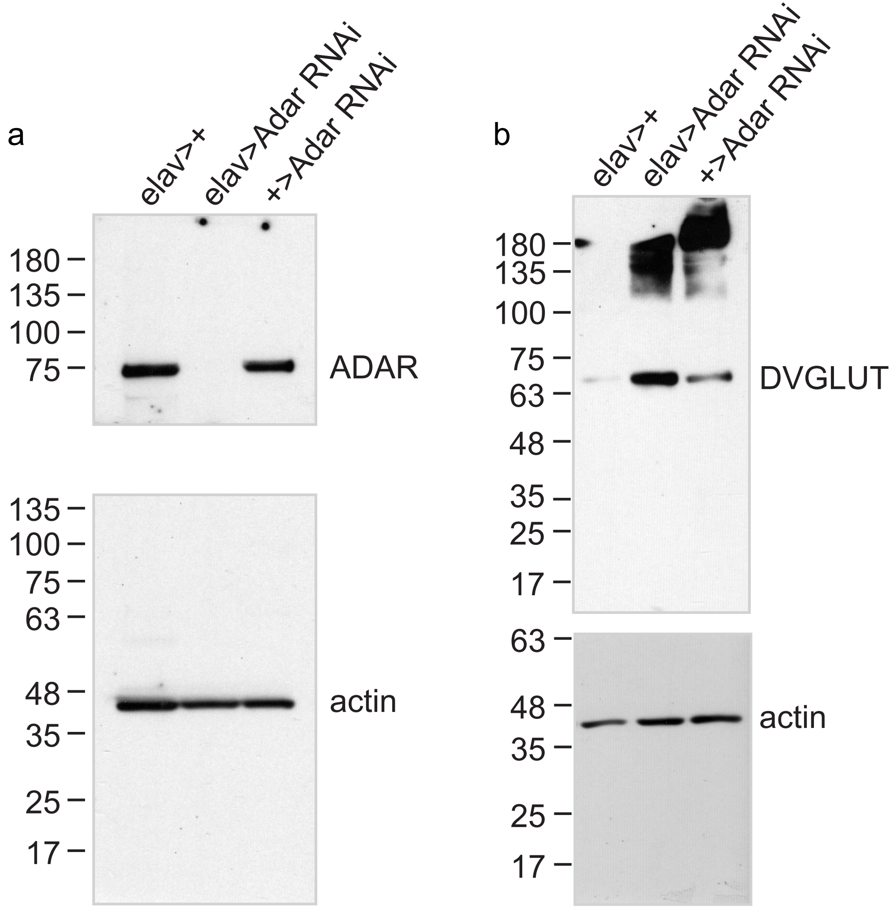

Supplemental Figure 2. Full length Western blots of ADAR and DVGLUT from *Adar* knockdown and control flies. (a) Efficient knockdown of ADAR expression is achieved in elav>*Adar* RNAi flies. (b) DVGLUT is elevated in elav>*Adar* RNAi relative to control animals.

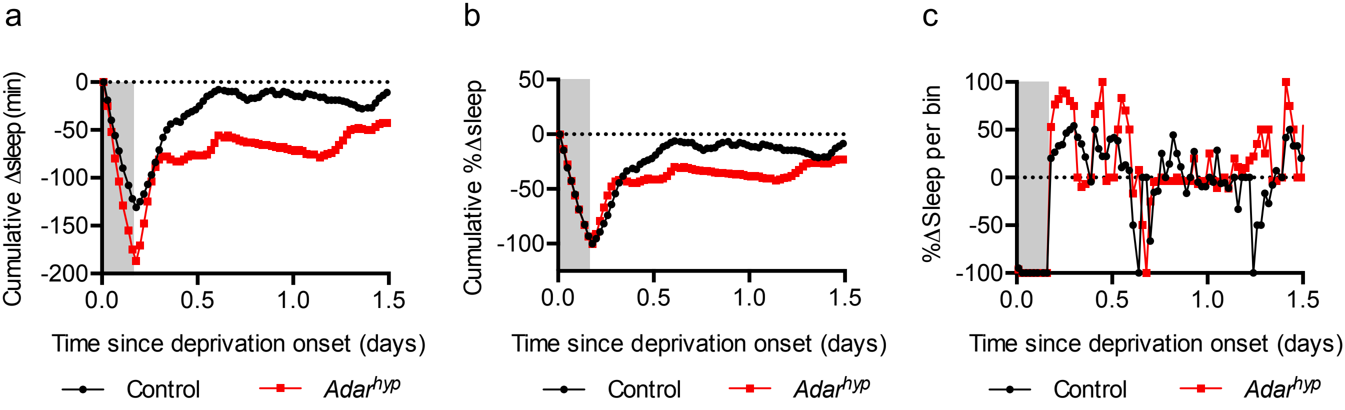

Supplemental Figure 3. Rate of change in sleep during deprivation and recovery periods. (a) Cumulative absolute and (b) cumulative relative changes in sleep show slower rate of recovery in *Adar^{hyp}* relative to control animals. (c) Percent change in sleep per bin trends higher for *Adar^{hyp}* than control animals. For all panels, the gray shaded region marks the sleep deprivation period.

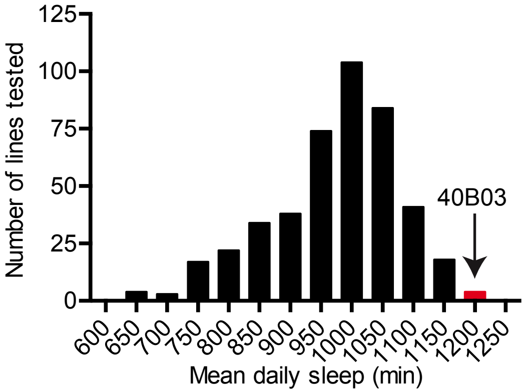

Supplementary Figure 4. Enhancer-GAL4 screen for *Adar*-mediated sleep-regulating neurons. Daily sleep was calculated for each Gal4>*Adar* RNAi combination and binned accordingly. The bin containing 40B03-Gal4 (arrow) is indicated in red.

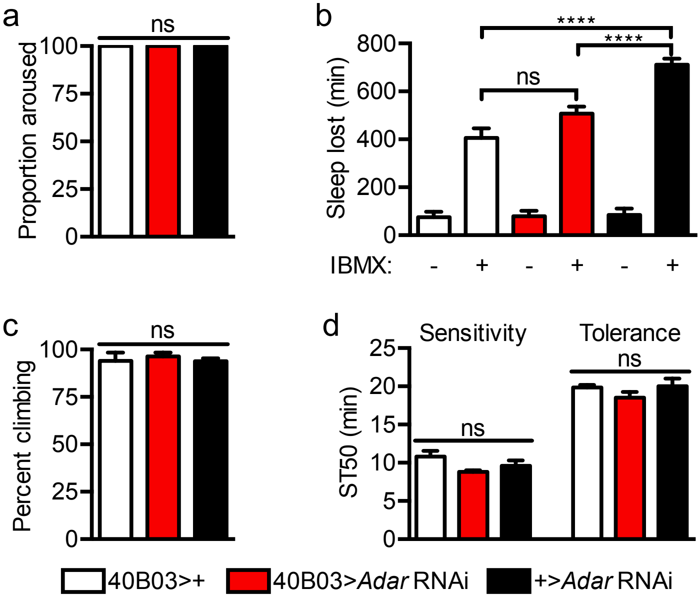

Supplementary Figure 5. Behavioral changes in 40B03>*Adar* RNAi animals are attributable to increased sleep. (a) Behavioral immobility in 40B03>*Adar* RNAi animals was acutely reversible by mechanical perturbation [n=3 trials per genotype; 8 animals were used for each trial]. (b) Behavioral immobility in 40B03>*Adar* RNAi animals was sup- pressible by the caffeine analog, IBMX [n=31 animals per genotype]. (c) Like controls, 40B03>*Adar* RNAi animals climbed 9.5 cm in 30 sec [n=3 trials of 10 flies per genotype]. (d) 40B03>*Adar* RNAi and control flies exhibited equivalent sensitivity and tolerance to ethanol [n=3 trials per genotype; 10 animals were used for each trial].

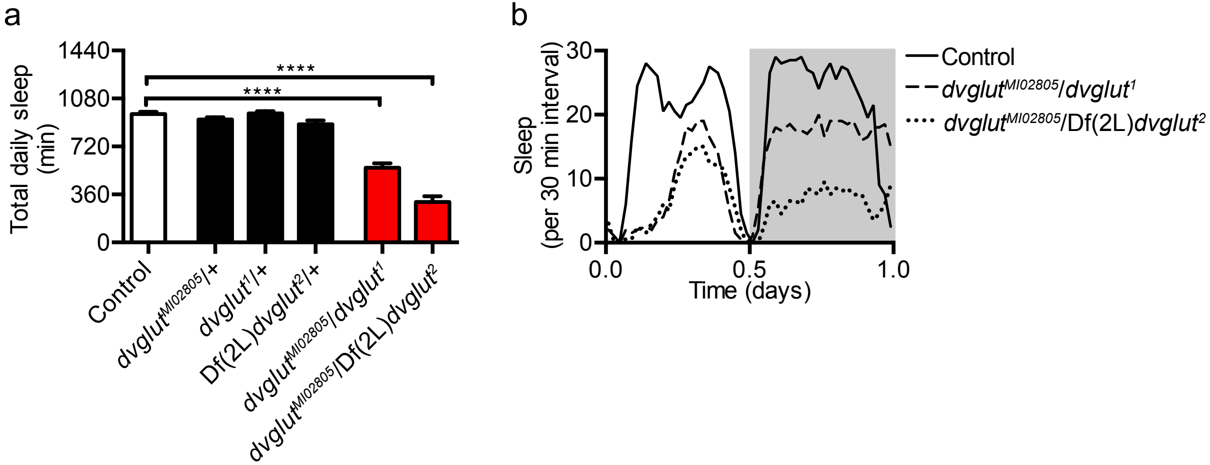

Supplementary Figure 6. Severe reduction in *dvglut* suppresses sleep. (a and b) Total daily sleep (a) and representative sleep profiles (b) of heteroallelic combinations of *dvglut* mutants and controls [n=22-31 for each genotype]. In this figure, control refers to animals harboring two copies of the wild-type *dvglut* allele.

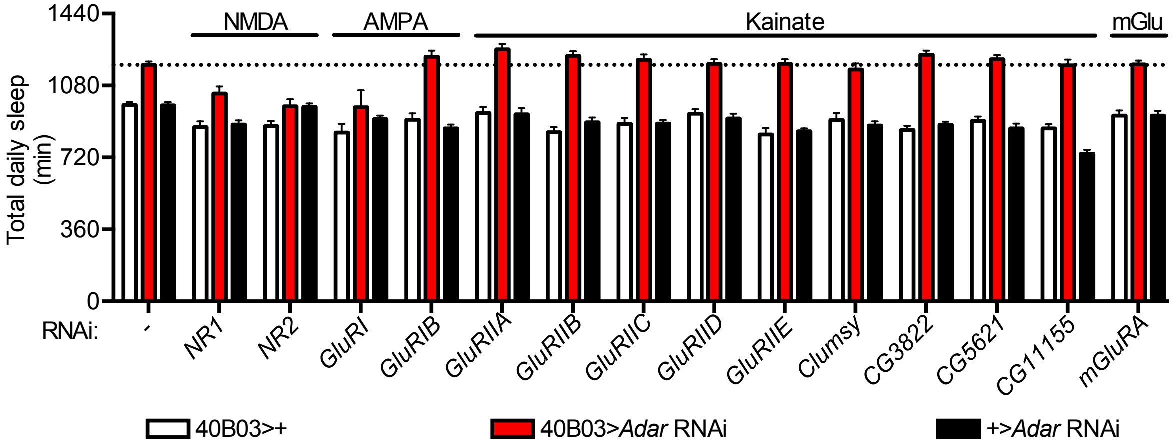

Supplementary Figure 7. Knockdown of NMDA and AMPA receptors restores sleep to *Adar*-deficient animals. Total daily sleep measured in control, 40B03>*Adar* RNAi, and 40B03>*Adar* RNAi in combination with RNAi's against ionotropic (NMDA, AMPA, kainate) and metabotropic (mGlu) glutamate receptors [n=7-31 for all genotypes].

Genotype	n	Total daily sleep mean (min)	Std. deviation	SEM
+>*Adar* RNAi	32	906	99	17.5
elav>+	31	944	92	16.5
elav>*Adar* RNAi	28	1299	61	11.6
c522>+	31	1022	103	18.4
c522>*Adar* RNAi	32	1000	102	18.1
104y>+	31	898	102	18.4
104y>*Adar* RNAi	32	789	120	21.2
cha>+	32	971	118	20.8
cha>*Adar* RNAi	31	805	115	20.7
sss>+	32	874	67	11.9
sss>*Adar* RNAi	32	893	107	19.0
30y>+	31	859	102	18.4
30y>*Adar* RNAi	32	852	116	20.6
TPH>+	32	932	79	13.9
TPH>*Adar* RNAi	32	954	108	19.0
201y>+	24	1061	78	15.9
201y>*Adar* RNAi	20	1046	97	21.8
Tdc2>+	32	983	98	17.3
Tdc2>*Adar* RNAi	31	918	107	19.3
TH>+	32	1005	88	15.6
TH>*Adar* RNAi	32	962	98	17.3
OK371>+	30	939	139	25.5
OK371>*Adar* RNAi	32	814	130	23.0
GAD>+	32	1028	81	14.2
GAD>*Adar* RNAi	32	958	81	14.3
Ddc>+	32	977	90	15.8
Ddc>*Adar* RNAi	29	874	94	17.5
MB247>+	25	865	157	31.4
MB247>*Adar* RNAi	32	742	138	24.4
tim>+	32	974	72	12.6
tim>*Adar* RNAi	25	925	116	23.2
clk-4.1m>+	32	980	84	14.8
clk-4.1m>*Adar* RNAi	28	876	112	21.2
cry>+	15	963	90	23.2
cry>*Adar* RNAi	11	962	103	30.9
24B>+	29	890	97	18.1
24B>*Adar* RNAi	29	778	125	23.1
OK107>+	15	922	106	27.2
OK107>*Adar* RNAi	16	810	81	20.3
npf>+	16	932	89	22.2
npf>*Adar* RNAi	16	770	123	30.6
pdf>+	15	841	111	28.7
pdf>*Adar* RNAi	36	824	184	30.6
vGlut>+	16	828	67	16.8
vGlut>*Adar* RNAi	16	806	79	19.7
trh>+	16	750	122	30.4
trh>*Adar* RNAi	16	836	126	31.5
ple>+	16	914	74	18.5
ple>*Adar* RNAi	16	897	97	24.3

Supplementary Table 1. Sleep effects of GAL4>*Adar* RNAi combinations using established, well-characterized drivers. Total number of animals tested (n), total daily sleep, standard deviation, and standard error of the mean (SEM) are listed for the various GAL4 lines used to knock down *Adar*.
